# Primary Cemented Bipolar Hemiarthroplasty Versus Proximal Femoral Nail Fixation for Unstable Intertrochanteric Fractures in Osteoporotic Elderly Patients: A Prospective Observational Study

**DOI:** 10.7759/cureus.114899

**Published:** 2026-08-20

**Authors:** Anurag Sharma, Manish Mehta, Shiv Prakash Sharma, Kaushlesh Sharma, Devkant Meena

**Affiliations:** 1 Orthopedics, Sawai Man Singh (SMS) Medical College, Jaipur, IND; 2 Preventive Medicine, Rajasthan University of Health Sciences (RUHS) College of Medical Sciences, Jaipur, IND

**Keywords:** bipolar hemiarthroplasty, functional outcome, harris hip score, intertrochanteric fracture, osteoporosis, proximal femoral nail

## Abstract

Introduction

Unstable intertrochanteric fractures in osteoporotic elderly patients are associated with substantial morbidity, delayed mobilization, and risk of fixation failure. The optimal surgical treatment remains debated. This study evaluated functional outcomes and implant-related complications in elderly patients with unstable intertrochanteric fractures treated with either primary cemented bipolar hemiarthroplasty (BHA) or proximal femoral nail (PFN) fixation.

Methods

This prospective observational study was conducted at a tertiary care orthopedic center between April 2022 and June 2025. Sixty patients aged 60-80 years with unstable intertrochanteric fractures (Evans types III and IV) were included. Patients underwent either cemented BHA (n=30) or PFN fixation (n=30) as determined by the treating surgical team. Primary outcome measures included time to full weight-bearing (FWB), Harris Hip Score (HHS) at one year, and revision surgery rate. Secondary outcomes included Parker-Palmer Mobility Score and limb length discrepancy (LLD).

Results

Patients treated with BHA achieved FWB significantly earlier than those treated with PFN (5.87±3.97 days vs. 12.83±1.62 days; p<0.001). At the one-year follow-up, the BHA group demonstrated significantly higher mean HHS (89.44±4.69 vs. 85.79±8.26; p=0.048) and Parker-Palmer Mobility Scores (8.15±0.95 vs. 7.38±0.86; p=0.002). Revision surgery was required in one patient (3.3%) in the BHA group and four patients (13.3%) in the PFN group. LLD greater than 10 mm was observed in three patients (10%) following BHA and in none of the PFN patients.

Conclusions

In this prospective observational study, patients managed with primary cemented BHA demonstrated earlier postoperative mobilization and better functional outcomes at one year compared with those treated with PFN fixation. These findings suggest that hemiarthroplasty may be a useful treatment option in selected elderly patients with unstable intertrochanteric fractures where rapid rehabilitation is a major treatment objective.

## Introduction

Intertrochanteric fractures are among the most common fragility fractures encountered in the elderly population and are associated with substantial morbidity, mortality, and healthcare utilization [1,2]. Increasing life expectancy and the growing prevalence of osteoporosis have contributed to a rising incidence of these injuries worldwide [2]. Unstable intertrochanteric fractures, particularly Evans type III and IV patterns, present a significant therapeutic challenge because comminution and poor bone quality can compromise fracture stability and increase the risk of fixation failure [3].

Internal fixation using a proximal femoral nail (PFN) is widely accepted as a standard treatment modality for intertrochanteric fractures. The biomechanical advantages of intramedullary fixation facilitate fracture stabilization while preserving the native hip joint. However, in elderly osteoporotic patients with unstable fracture configurations, complications such as implant cut-out, fixation failure, excessive fracture collapse, and delayed mobilization remain important concerns [3,4]. Furthermore, prolonged protected weight-bearing may predispose patients to pressure ulcers, respiratory infections, venous thromboembolism, and functional decline [5].

Primary bipolar hemiarthroplasty (BHA) has emerged as an alternative treatment option for selected elderly patients with unstable intertrochanteric fractures. By replacing the fractured proximal femur and providing immediate mechanical stability, hemiarthroplasty facilitates early mobilization and unrestricted weight-bearing [6,7]. Previous studies have reported favorable functional outcomes and lower rates of mechanical failure with primary arthroplasty in osteoporotic patients with unstable fracture patterns [6-9].

Despite the increasing use of hemiarthroplasty for unstable intertrochanteric fractures, the optimal surgical management of these injuries remains controversial. Both PFN fixation and BHA have distinct advantages and limitations, and comparative evidence regarding functional recovery, postoperative mobility, and implant-related complications continues to evolve [4,6-9].

Therefore, the present prospective observational study was undertaken to evaluate and compare functional outcomes, postoperative mobility, and implant-related complications among elderly patients with unstable intertrochanteric fractures managed with either primary cemented BHA or PFN fixation.

## Materials and methods

Study design and setting

This prospective observational study was conducted in the Department of Orthopedics of Sawai Man Singh (SMS) Medical College, a tertiary care teaching hospital in Jaipur, India, between April 2022 and June 2025. Ethical approval was obtained from the Ethics Committee of Sawai Man Singh (SMS) Medical College and Attached Hospitals (approval number: 372 MC/EC/2023). The study protocol was reviewed and approved by the committee on January 18, 2024. Written informed consent was obtained from all participants prior to enrollment.

Study population

Patients aged 60-80 years presenting with unstable intertrochanteric fractures of the femur (Evans types III and IV) and considered fit for surgical intervention were included in the study. Patients with pathological fractures, polytrauma, open fractures, or previous surgery involving the ipsilateral hip or those unwilling to participate were excluded. The sample size was calculated based on the primary outcome measure, the Harris Hip Score (HHS). Assuming a minimum clinically significant difference of 4 points between the groups, a standard deviation of 5 points, a power of 80%, and a two-sided alpha error of 5%, the minimum required sample size was estimated to be 25 patients per group using OpenEpi (Dean AG, Sullivan KM, Soe MM; OpenEpi: Open Source Epidemiologic Statistics for Public Health, Version 3.01; www.OpenEpi.com, updated 2013/04/06). To compensate for potential loss to follow-up, 30 patients were included in each group, resulting in a total sample size of 60 participants. Hence, a total of 60 eligible patients were enrolled and followed prospectively for a minimum period of one year.

Surgical procedure

**Figure 1:**
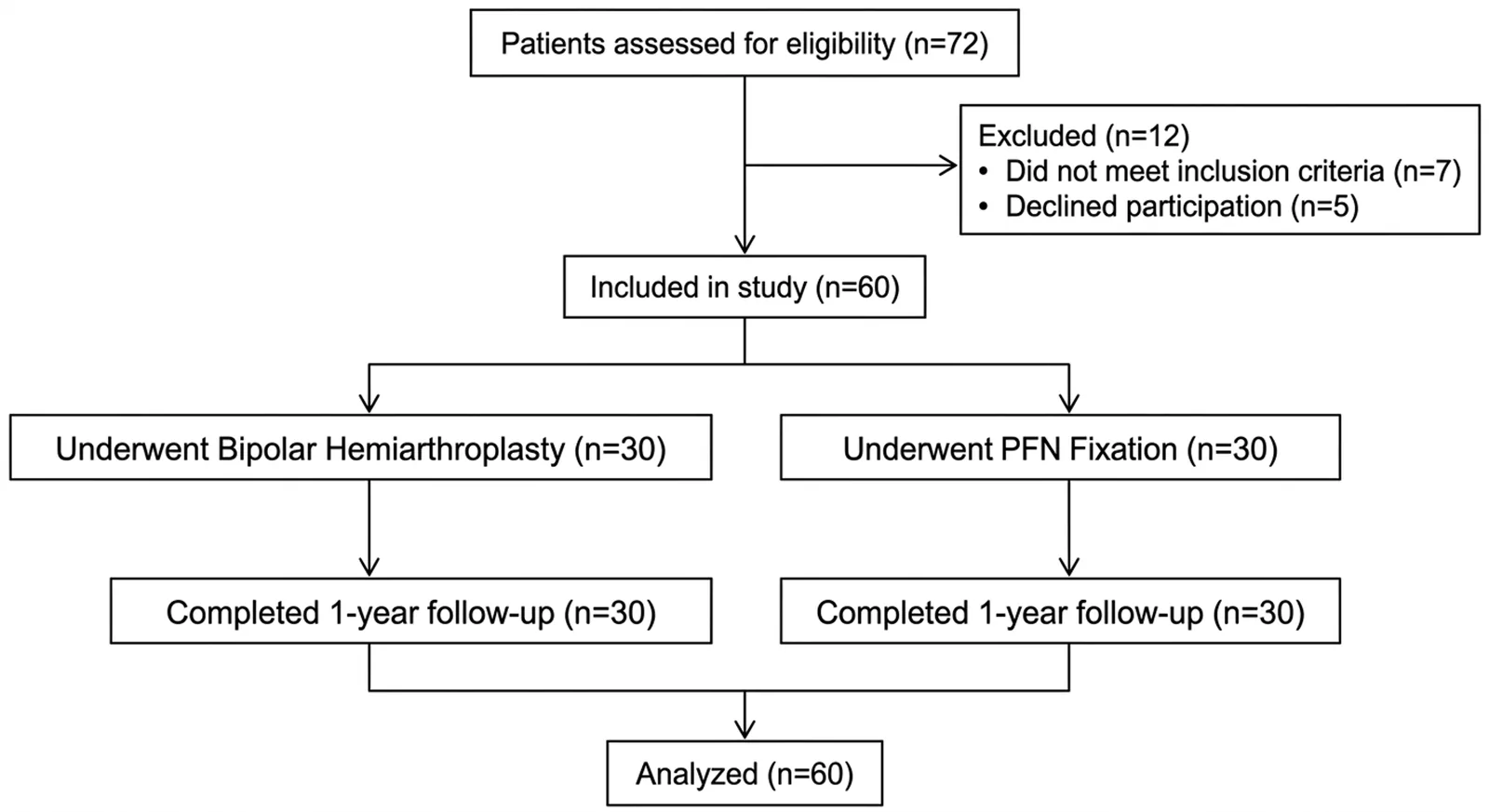
STROBE flow diagram illustrating patient screening, eligibility assessment, inclusion, treatment allocation, follow-up, and final analysis in the study cohort STROBE: Strengthening the Reporting of Observational Studies in Epidemiology; PFN: proximal femoral nail

The choice of surgical treatment was determined by the treating orthopedic surgeon based on fracture configuration, bone quality, patient characteristics, and intraoperative considerations. Thirty patients underwent primary cemented BHA, while 30 patients underwent fixation with a PFN. In the BHA group, surgery was performed through a standard posterior approach using a cemented bipolar prosthesis. In the PFN group, fracture fixation was carried out using a PFN according to standard operative techniques. Patient recruitment, eligibility assessment, follow-up, and final analysis are summarized in the Strengthening the Reporting of Observational Studies in Epidemiology (STROBE) flow diagram (Figure 1).

Postoperative rehabilitation

Patients who underwent hemiarthroplasty were mobilized with walker-assisted full weight-bearing (FWB) as tolerated beginning on the third postoperative day. Patients treated with PFN commenced knee mobilization and quadriceps strengthening exercises immediately after surgery. Progression to FWB was guided by fracture stability and clinical assessment and generally occurred between 10 and 15 days postoperatively.

Outcome measures

Functional outcome was assessed using the HHS described by Harris [10] and the Parker-Palmer Mobility Score described by Parker and Palmer [1]. Both assessment instruments are published clinical scoring systems available in the public domain for academic and research use and do not require permission or licensing for utilization in the present study.

The primary outcome measures included time to FWB, HHS at one-year follow-up, and requirement for revision surgery. Secondary outcome measures included Parker-Palmer Mobility Score and limb length discrepancy (LLD) greater than 10 mm at the final follow-up.

Statistical analysis

Data were entered into Microsoft Excel (Microsoft Corporation, Redmond, Washington, United States) and analyzed using IBM SPSS Statistics for Windows, Version 23.0 (IBM Corp., Armonk, New York, United States). Continuous variables were expressed as mean±standard deviation and compared using the independent samples t-test. Categorical variables were expressed as frequencies and percentages and compared using the chi-squared test or Fisher's exact test wherever appropriate. A p-value of less than 0.05 was considered statistically significant.

## Results

A total of 60 patients with unstable intertrochanteric fractures were included in the study, with 30 patients undergoing primary cemented BHA and 30 patients undergoing PFN fixation. All patients completed a minimum follow-up period of one year and were available for final functional assessment.

Functional outcomes

Patients treated with BHA achieved FWB significantly earlier than those treated with PFN fixation. The mean time to FWB was 5.87±3.97 days in the BHA group compared with 12.83±1.62 days in the PFN group (p<0.001).

At the one-year follow-up, the mean HHS was significantly higher in the BHA group than in the PFN group (89.44±4.69 vs. 85.79±8.26; p=0.048). Similarly, the mean Parker-Palmer Mobility Score was significantly greater among patients treated with BHA (8.15±0.95) compared with those treated with PFN fixation (7.38±0.86; p=0.002). Detailed functional outcome measures are presented in Table 1.

**Table 1:** Comparison of functional recovery outcomes *Unpaired t-test BHA: bipolar hemiarthroplasty; PFN: proximal femoral nail; FWB: full weight-bearing; HHS: Harris Hip Score

Outcome		BHA group (n=30)	PFN group (n=30)	P-value
Time to FWB (days)	Mean±SD	5.87±3.97	12.83±1.62	<0.001*
Final HHS	Mean±SD	89.44±4.69	85.79±8.26	0.048*
Final Parker-Palmer Score	Mean±SD	8.15±0.95	7.38±0.86	0.002*

Implant survival and complications

Revision surgery was required in one patient (3.3%) in the BHA group and four patients (13.3%) in the PFN group. The indication for revision in the BHA group was deep infection, whereas all revision procedures in the PFN group were performed for implant cut-out and fixation failure.

LLD greater than 10 mm was observed in three patients (10%) in the BHA group, including two cases of shortening and one case of lengthening. No patient in the PFN group developed a clinically significant LLD. Details regarding implant-related complications are summarized in Table 2.

**Table 2:** Implant survival and related complications *Fisher's exact test BHA: bipolar hemiarthroplasty; PFN: proximal femoral nail

Parameter	BHA group (n=30)	PFN group (n=30)	P-value
Revision surgery, n (%)	1 (3.33%)	4 (13.33%)	0.161*
Limb length discrepancy, n (%)	3 (10%)	0 (0%)	0.237*

## Discussion

The management of unstable intertrochanteric fractures in elderly osteoporotic patients remains a subject of ongoing debate. While PFN fixation is widely accepted as a standard treatment modality, concerns regarding fixation failure, prolonged protected weight-bearing, and delayed functional recovery have prompted increasing interest in primary arthroplasty for selected patients. The present prospective observational study compared functional outcomes and implant-related complications among elderly patients managed with either primary cemented BHA or PFN fixation.

A notable finding of the present study was the significantly earlier achievement of FWB in the BHA group compared with the PFN group. The ability to mobilize patients early following hemiarthroplasty is one of its principal theoretical advantages and may reduce complications associated with prolonged immobilization in elderly individuals. Similar observations have been reported by Haentjens et al. and Kim et al., who demonstrated that arthroplasty facilitates early ambulation and functional recovery in patients with unstable intertrochanteric fractures [6,9].

Patients treated with BHA also demonstrated significantly higher HHS and Parker-Palmer Mobility Scores at the one-year follow-up. These findings suggest that early restoration of mobility may translate into improved functional outcomes during the postoperative period. Previous studies by Chan and Gill, Grimsrud et al., and Kim et al. have similarly reported favorable functional results following hemiarthroplasty in osteoporotic patients with unstable fracture patterns [7-9]. Nevertheless, it is noteworthy that both treatment groups achieved generally satisfactory functional outcomes, indicating that both procedures remain viable treatment options when appropriately selected.

Although revision surgery was observed more frequently in the PFN group, the relatively small number of events limits definitive conclusions regarding implant survival. All revisions in the PFN group were related to implant cut-out and fixation failure, complications that have been well documented in osteoporotic bone and unstable fracture configurations [3,11]. In contrast, the single revision in the BHA group was attributable to deep infection rather than mechanical failure. These findings are consistent with previous reports suggesting that arthroplasty may reduce the risk of mechanical complications associated with fixation in poor-quality bone [6-8].

LLD greater than 10 mm was observed only in the BHA group. This finding highlights a recognized technical challenge of hemiarthroplasty performed for fracture management, where normal anatomical landmarks may be disrupted by fracture comminution. Careful preoperative planning and meticulous intraoperative assessment remain important to minimize this complication. Recent systematic reviews and meta-analyses have demonstrated that arthroplasty facilitates earlier FWB and improved early functional recovery in elderly patients with unstable intertrochanteric fractures. However, these studies have generally not demonstrated consistent superiority with respect to long-term functional outcomes, mortality, or reoperation rates when compared with PFN fixation [12,13].

The findings of the present study suggest that primary cemented BHA may be particularly beneficial in elderly patients with unstable intertrochanteric fractures who require early mobilization and are at increased risk of complications related to prolonged immobilization. However, treatment decisions should remain individualized and take into account patient characteristics, fracture morphology, bone quality, functional demands, and surgeon experience.

Limitations

This study has several limitations. First, the sample size was relatively small, which may have limited the ability to detect significant differences in uncommon complications. Second, treatment allocation was not randomized because of the observational study design, introducing the possibility of selection bias. Third, the study was conducted at a single center, which may limit the generalizability of the findings. Finally, the follow-up period of one year was adequate for the assessment of functional recovery and early complications but insufficient for evaluating long-term outcomes such as prosthetic loosening, acetabular erosion, or late implant failure.

Despite these limitations, the prospective design, complete follow-up of all enrolled patients, and use of validated functional outcome measures strengthen the findings of the study and contribute to the growing evidence regarding the management of unstable intertrochanteric fractures in elderly patients.

## Conclusions

In this prospective observational study, primary cemented BHA was associated with earlier postoperative mobilization and better functional outcomes at the one-year follow-up compared with PFN fixation in elderly patients with unstable intertrochanteric fractures. Although revision surgery was observed less frequently in the hemiarthroplasty group, the small number of events precludes definitive conclusions regarding implant survival. LLD was encountered only among patients treated with hemiarthroplasty, highlighting the importance of meticulous surgical technique. Overall, the findings suggest that primary cemented BHA may represent a valuable treatment option for selected elderly patients with unstable intertrochanteric fractures, particularly when rapid rehabilitation and early return to mobility are important treatment goals. Further multicenter studies with larger sample sizes and longer follow-up are required to confirm these findings.
